# The effects of ventilation on left-to-right shunt and regional cerebral oxygen saturation: a self-controlled trial

**DOI:** 10.1186/s12871-019-0852-1

**Published:** 2019-10-09

**Authors:** Peiyi Li, Jun Zeng, Wei Wei, Jing Lin

**Affiliations:** 1 Institute of Hospital Management, West China Hospital, Sichuan University, Guo Xue Xiang 37, Chengdu, Sichuan China; 2 Department of Anesthesiology, West China Hospital, Sichuan University, Guo Xue Xiang 37, Chengdu, 610041 Sichuan China

**Keywords:** Arterial partial pressure of carbon dioxide tension, Left-to-right shunt, Pulmonary vascular resistance, Ventilation strategy

## Abstract

**Background:**

Increase of pulmonary vascular resistance (PVR) is an efficient method of modulating pulmonary and systemic blood flows (Qp/Qs) for patients with left-to-right (L-R) shunt, and is also closely associated with insufficient oxygen exchange for pulmonary hypoperfusion. So that it might be a preferred regime of maintaining arterial partial pressure of carbon dioxide tension (PaCO_2_) within an optimal boundary via ventilation management in congenital heart disease (CHD) patients for the inconvenient measure of the PVR and Qp/Qs. However, the appropriate range of PaCO_2_ and patient-specific mechanical ventilation settings remain controversial for CHD children with L-R shunt.

**Methods:**

Thirty-one pediatric patients with L-R shunt, 1–6 yr of age, were included in this observation study. Patients were ventilated with tidal volume (V_T_) of 10, 8 and 6 ml/kg in sequence, and 15 min stabilization period for individual V_T_. The velocity time integral (VTI) of L-R shunt, pulmonary artery (PA) and descending aorta (DA) were measured with transesophageal echocardiography (TEE) after an initial 15 min stabilization period for each V_T_, with arterial blood gas analysis. Near-infrared spectroscopy sensor were positioned on the surface of the bilateral temporal artery to monitor the change in regional cerebral oxygen saturation (rScO_2_).

**Results:**

PaCO_2_ was 31.51 ± 0.65 mmHg at V_T_ 10 ml/kg vs. 37.15 ± 0.75 mmHg at V_T_ 8 ml/kg (*P* < 0.03), with 44.24 ± 0.99 mmHg at V_T_ 6 ml/kg significantly higher than 37.15 ± 0.75 mmHg at V_T_ 8 ml/kg. However, PaO_2_ at a V_T_ of 6 ml/kg was lower than that at a V_T_ of 10 ml/kg (*P* = 0.05). Meanwhile, 72% (22/31) patients had PaCO_2_ in the range of 40-50 mmHg at V_T_ 6 ml/kg. VTI of L-R shunt and PA at V_T_ 6 ml/kg were lower than that at V_T_ of 8 and 10 ml/kg (*P* < 0.05). rScO_2_ at a V_T_ of 6 ml/kg was higher than that at a V_T_ of 8 and 10 ml/kg (*P* < 0.05), with a significantly correlation between rScO_2_ and PaCO_2_ (r = 0.53). VTI of PA in patients with defect diameter > 10 mm was higher that that in patients with defect diameter ≤ 10 mm.

**Conclusions:**

Maintaining PaCO_2_ in the boundary of 40-50 mmHg with V_T_ 6 ml/kg might be a feasible ventilation regime to achieve better oxygenation for patients with L-R shunt. Continue raising PaCO_2_ should be careful.

**Trail registration:**

Clinical Trial Registry of China (http://www.chictr.org.cn) identifier: ChiCTR-OOC-17011338, prospectively registered on May 9, 2017.

## Background

Pulmonary oxygen exchange and cardiac output (CO) are closely associated with adequate tissue oxygenation, which could be evaluated by the ratio of pulmonary and systemic blood flow (Qp/Qs) [1]. In congenital heart disease (CHD) children with left-to-right (L-R) shunt, the ratio of Qp/Qs often more than one due to the steal of pulmonary blood flow from systemic blood flow [2], and result in pulmonary hyperperfusion and poor systemic perfusion, which was associated with seriously complications, including pulmonary hemorrhage and necrotizing enterocolitis [3].

Increasing pulmonary vascular resistance (PVR) is a double-blade sword, since it would not only augment right ventricular afterload and lessen L-R shunt [4], but also lead to the insufficient pulmonary oxygenation and deteriorate tissue oxygenation [5]. Therefore, the key regime of achieving better oxygenation in patients with L-R shunt is to balance the PVR for a favorable ratio of Qp/Qs [6, 7]. However, precisely measuring PVR and Qp/Qs is complicated and time-consuming in the operating room. In Reddy’s study, increased PVR, CO and reduced ratio of Qp/Qs through enhancing PaCO_2_ was testified [8], similar clinical phenomenon was also observed after 4 % CO_2_ added to the fresh gas flow in a 6-year-old patient with a 4 mm Blalock-Taussing shunt [9]. These indicated that PaCO_2_ which could be noninvasively measured via blood gases is more likely to be an indicator of unstable PVR and Qp/Qs [10]. However, the definite PaCO_2_ level that would cause intended change in L-R shunt at patient with congenital heart lesions remain unknown. Besides, adjusting PaCO_2_ by ventilation management is preferred by anesthesiologists for its available and handy, compare to adding CO_2_ to the fresh gas. Whereas, how to maintain a favorable level of PaCO_2_ by regulating mechanical ventilation parameters is still unclear, and largely derived from anesthesiologist’s personal experience to alleviate this unequal Qp/Qs distribution in patients with L-R shunt.

The aim of our study was to compare the VTI of L-R shunt, PA and DA blood flow, cerebral oxygen saturation (rScO_2_) by interfering with common mechanical ventilation parameters for pediatrics: V_T_ 10, 8 and 6 ml/kg respectively. We attempted to find a perioperative ventilation strategy with a proper range of PaCO_2_ which could provide an appropriate Qp/Qs with optimal oxygen supply for children with L-R shunt.

## Methods

### Patient

This study was approved by Ethics Committee of West China Hospital of Sichuan University and then registered on Clinical Trial Registry of China (ChiCTR-OOC-17011338). Eligible subjects were clinically stable children of ASA II-III and aged 1-6 years with a diagnosis of L-R shunt confirmed by echocardiography and scheduled for elective cardiac surgery. Written informed consent from parents or legal guardians was obtained. Children with pulmonary diseases, heart failure or severe arrhythmia were excluded. The case would be cancelled if had one of the followings: difficult intubation, bronchial spasm and unfinished experiment before cardiopulmonary bypass.

A standardized anesthetic protocol was administered. Electrocardiogram (ECG), peripheral oxygen saturation (SpO_2_) and mean blood pressure (MAP) were performed on arrival at the operating room. General anesthesia was induced with midazolam (0.2~0.3 mg/kg), sufentanil (1~1.5 μg/kg) and rocuronium (0.6~1 mg/kg). After intubated a tracheal tube, bilateral lung ventilations were evaluated by auscultation. Then patients were meachanically ventilated with volume control mode (Aisys CS^2^, Datex-Ohmeda, WI, USA). Initial settings were V_T_ 10 ml/kg, ratio of inspiratory to expiratory at 1:2 and FiO_2_ at 0.6, with ventilator rate adjusted according to age (1 to 3 years old: 20–25 rates/min, 3 to 6 years old: 16–20 rates/min). After induction of anesthesia, a radial arterial catheter was inserted for invasive arterial blood pressure monitoring and gas sampling. During next procedure, end-tidal carbon dioxide partial pressure (EtCO_2_) and invasive arterial pressure (IAP) were monitored continuously. In addition, two sensors of cerebral oximeter (EGOS-600, Enginmed, Suzhou, China) were placed bilaterally on forehead to detect rScO_2_ [11].

Anesthesia was maintained with 1~2% sevofurane, additional rocuronium and sufentanil were given intravenously when necessary. Moreover, all patients received 10~15 ml/kg of crystalloid in the first hour. Core body temperature remained stable at 36° to 37 °C throughout the study.

### Experiment protocol

After intubation, all children were ventilated in three level of V_T_ in the order of 10 ml/kg, 8 ml/kg and 6 ml/kg. Each V_T_ level was maintained for 15 min. TEE examinations and blood gas measurement were performed after 15 min of stabilization ventilation at each V_T_ level. Data of rScO_2_ was automatic recording every 3 s during the operation and downloaded to a storage disk for further analysis after surgery.

### TEE measurements

TEE, regarding as a reliable and minimally invasive monitoring technology, had been used in cardiac surgery [12]. Descending aorta blood flow (DA) which occupies almost 70% of CO could reflect the change in systemic blood flow. The velocity time integral (VTI) of L-R shunt, pulmonary artery (PA) and DA were measured respectively to represent blood flow assuming that the diameters of arteries changed subtly than arterioles by PaCO_2_ [13, 14].

**Fig. 1:**
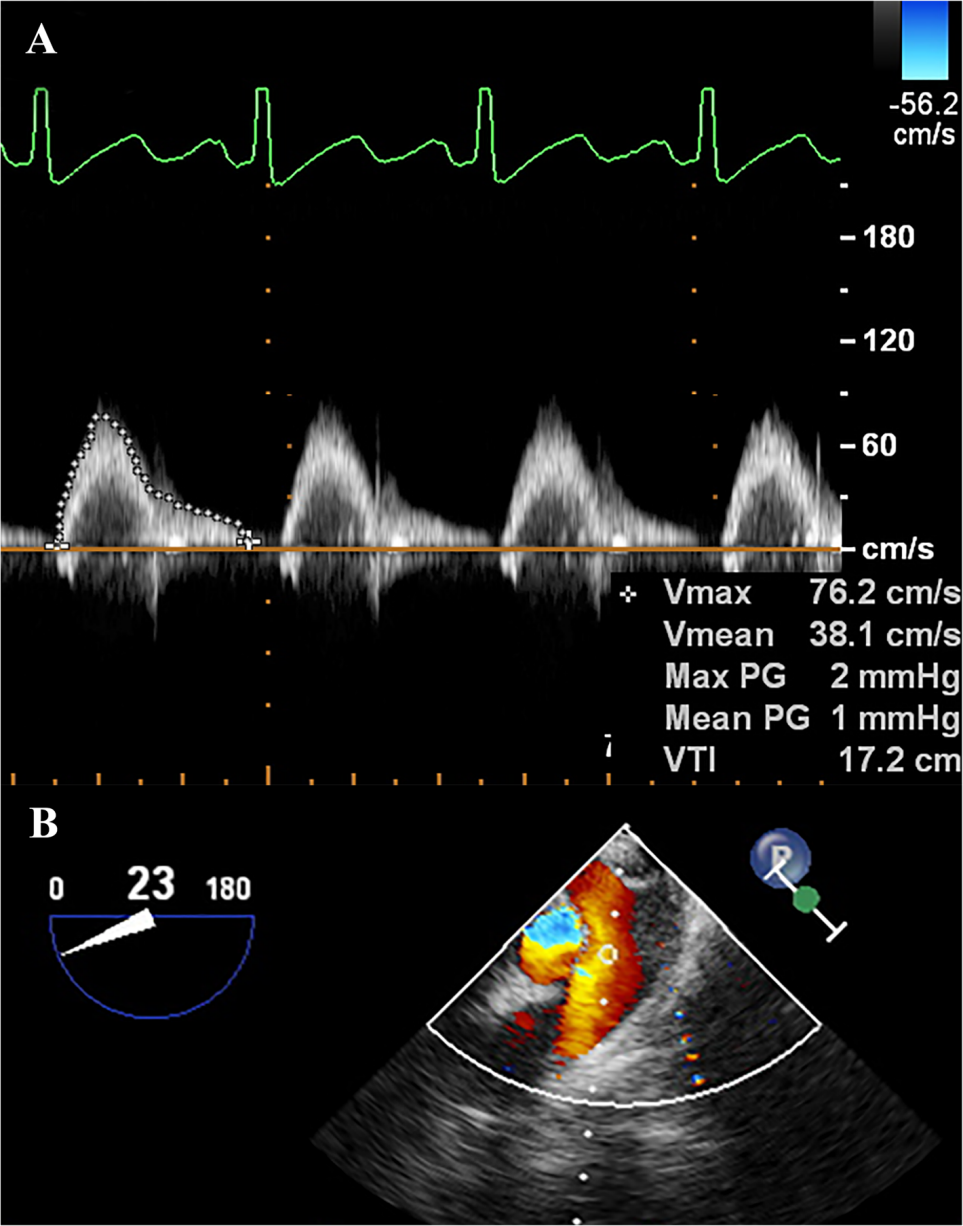
A 11-year-old patient diagnosed with VSD. VTI of PA were measured respectively at short axis of ascending aorta (**a**, **b**)

Detailed TEE measurements as follows: a TEE probe was inserted after intubation, and connected to an ultrasound system (Philips iE33, Bothell, WA, USA). M-line, color flow and spectral Doppler were used to measure the VTI of blood flow, assuring the angel between beam M-line and flow smaller than 20°. Once 15 min ventilation of V_T_ 10 ml/kg completed, the L-R shunt and PA flows were obtained at optimal TEE views, such as mid-esophageal (ME) four chamber and ME right ventricle inflow-outflow. The blood flow of DA was acquired via long-axis view of ascending aorta with the depth of TEE probe kept at mid-esophageal (ME) [15]. After 15 min of stabilization at following V_T_ level, the identical TEE views with V_T_ 10 ml/kg were scanned to acquire the VTI in each patient. All VTI of L-R shunt, PA and DA flows were traced and averaged over three consecutive cardiac cycles (Fig. 1a, b). All echocardiography recordings were obtained and analyzed by the same manipulator.

### Statistical analysis

A preliminary test of 5 patients revealed that at least 24 children were needed to detect a significant difference among three V_T_ assuming α = 0.05 and ß = 0.8. Data were tested for distribution and presented as mean ± SD. Differences among three V_T_ were analyzed using Related-Samples Wilcoxon Signed Rank test. The differences of between-group were evaluated by Student’s t-test. Besides, Spearman correlation test was performed to access correlations. *P* value of < 0.05 was considered statistically significant. All statistical analyses were performed with IBM SPSS Statistics 24 (SPSS, Inc., IL, USA).

## Results

All enrolled patients had completed and 93 TEE measurements was successfully obtained (Table 1). There was a statistically significant increase in PaCO_2_ and rScO_2_ as V_T_ progressive decreased from 10 ml/kg to 8 ml/kg and 6 ml/kg (Table 2). Low PaCO_2_ (below than 30 mmHg) occurred in 11/31 patients at V_T_ 10 ml/kg, and 5/31 patients occurred high PaCO_2_ (above than 50 mmHg) at V_T_ 6 ml/kg. In addition, the proportion of PaCO_2_ between 40 and 50 mmHg increased from 0% in V_T_ 10 ml/kg to 34% in V_T_ 8 ml/kg, and 72% among the V_T_ 6 ml/kg.

**Table 1:** Patients’ preoperative characteristics and operative details

Age (month)	33.52 ± 3.44
Weight (kg)	13.01 ± 0.79
Height (cm)	90.42 ± 4.0
Sex (Male/Female)	17/14
Type of defect (PDA/ASD/VSD)	1/5/25
Diameter of defect (mm)	9.81 ± 1.26
Length of surgery (hour)	3.51 ± 0.31

*ASD* Atrial septal defect, *PDA* Patent ductus arteriosus, *VSD* Ventricular septal defect

**Table 2:** Hemodynamics and other parameters with V_T_ 10, 8 and 6 ml/ kg

	V_T_ 10 ml/ kg	V_T_ 8 ml/ kg	V_T_ 6 ml/ kg
IAP (mmHg)	65.35 ± 2.15	62.39 ± 2.11	61.23 ± 2.52
HR (beats/min)	107.81 ± 3.45	105.58 ± 3.07	114.26 ± 3.01
VTI _PA_/VTI _DA_	1.83 ± 0.14	1.80 ± 0.15	1.71 ± 0.13
rScO_2_ (%)	67.23 ± 4.72	67.55 ± 4.54	69.34 ± 4.29*^#^
EtCO_2_ (mmHg)	30.87 ± 0.52	36.68 ± 0.69*	44.45 ± 1.06*^#^
PaCO_2_ (mmHg)	31.51 ± 0.65	37.15 ± 0.75*	44.24 ± 0.99*^#^
PaO_2_ (mmHg)	216.89 ± 10.72	214.89 ± 9.87	203.74 ± 8.97*

*EtCO*_*2*_ End-tidal carbon dioxide partial pressure, *HR* Heart rate, *IAP* Invasive arterial pressure, *PaCO*_*2*_ Arterial partial pressure of carbon dioxide tension, *PaO*_*2*_ Arterial oxygen pressure, *rSCO*_*2*_ Regional cerebral oxygen saturation, *V*_*T*_ Tidal volume

*P < 0.05 Respect to V_T_ 10 ml/kg; ^#^ *P* < 0.05 Respect to V_T_ 8 ml/kg

**Fig. 2:**
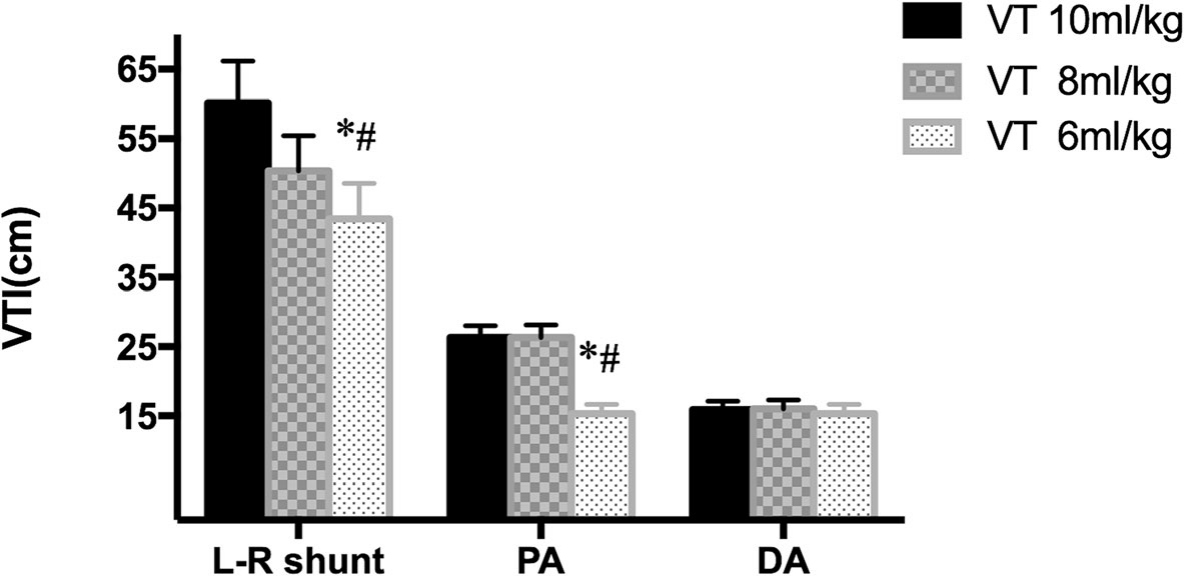
Changes in VTI of L-R shunt, PA and DA with V_T_ 10, 8 and 6 ml/kg. * *P* < 0.05 respect to V_T_ 10 ml/kg, ^#^ *P* < 0.05 respect to V_T_ 8 ml/kg

Figure 2 delineated a significantly stepwise decline in VTI of L-R shunt (from 60.16 ± 6.0 cm to 50.35 ± 5.12 cm and 43.44 ± 5.11 cm). Meanwhile, the corresponding VTI of PA markedly descended from 26.36 ± 1.68 cm to 26.33 ± 1.82 cm and 23.23 ± 1.55 cm with V_T_ 6 ml/kg compared to 10 and 8 ml/kg. Nevertheless, no obvious changes was found in the VTI of DA flow. Concurrently, the hypoventilation also resulted in a relatively slight but consistent reduction in the ratio of VTI _PA_/VTI _DA_ (Table 2).

**Fig. 3:**
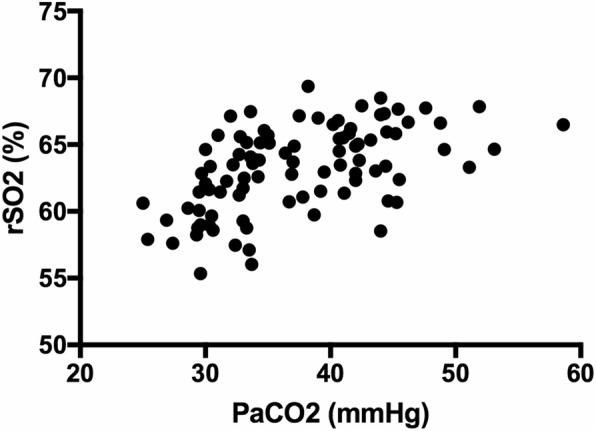
There was significant correlations between rScO_2_ and PaCO_2_ (r = 0.53, *P* = 0.03)

The mean value of VTI_PA_/VTI_DA_ was lower in the group of PaCO_2_ higher than 40 mmHg (1.85 vs 1.67), but without statistical significance. Meanwhile, the rScO_2_ risen 2.78% significantly following PaCO_2_ higher than 40 mmHg (from 62.14 to 64.92%), with a significant linear correlation between PaCO_2_ and rScO_2_ (r = 0.53) depicted in Fig. 3.

Children with defect> 10 mm were younger, accompanied by higher VTI of PA flow and longer hospitalization days (Table 3). In addition, the decrease of L-R shunt caused by rising PaCO_2_ was more striking in children whose defect≤10 mm rather than children with defect> 10 mm, although without significant difference.

**Table 3:** Differences between different defect diameters

	Defect diameter ≤ 10 mm	Defect diameter > 10 mm
Number of patients	20	11
Age (month)	36.19 ± 3.89	23.82 ± 3.56*
Weight (kg)	12.95 ± 0.99	11.88 ± 1.37
Height (cm)	92.67 ± 3.89	84.18 ± 3.57
VTI of L-R shunt (cm)	58.02 ± 5.36	39.12 ± 10.34
VTI of PA (cm)	22.86 ± 6.26	30.54 ± 3.16*
VTI of DA (cm)	15.01 ± 5.68	18.86 ± 2.73
ΔVTI of L-R shunt (cm)	19.74 ± 4.14	12.44 ± 3.9
VTI _PA_/VTI _DA_	1.75 ± 0.67	1.93 ± 0.2
rSCO_2_ (%)	63.66 ± 2.26	61.89 ± 0.51
Hospitalization stay (day)	9.95 ± 5.01	16.55 ± 2.15*

ΔVTI which indicated the absolute decrease of L-R shunt, was calculated by the absolute difference between the maximum and minimum of L-R shunt among three V_T_

* *P* < 0.05 respect to patients with defect diameter smaller than 10mm

## Discussion

In the present study, hyperventilation with V_T_ 10 ml/kg aggravated the VTI of L-R shunt and reduced rScO_2_ for pediatric patients with CHD, inversely, hypoventilation with V_T_ 6 ml/kg evoked moderate hypercapnia indicated with mitigating the excessive L-R shunt and raising rScO_2_.

Hyperventilation has traditionally been preferred to improve pulmonary oxygen exchange and reduce the incidence of perioperative desaturation. However, it may be harmful for CHD children with L-R shunt as the fragile balance of Qp/Qs might be exacerbated by decrease of PaCO_2_ [16, 17]. Seriously, the increase of Qp/Qs may result in imbalance of oxygen supply and postoperative mortality [18, 19]. CO_2_ could increase the extracellular concentration of Ca^2+^, constrict pulmonary arterioles and lead to an increase of PVR [20], which is likely to result in a reduction in L-R shunt and redistribute the blood from PA to the systemic blood flow. In this study, we founded the VTI of L-R shunt decreased by 27.8% as PaCO_2_ increased from 31 mmHg to 44 mmHg, meanwhile the VTI of PA decreased by 11.9%, without significant increase in the DA blood flow and MAP. The possible explanation was that the DA blood flow represented about 70% of CO, which may underestimate the slightly increase of systemic blood flow.

Previous studies demonstrated that adjusting Qp/Qs via ventilation strategies was an effective method in children with intra-cardiac shunt [21, 22]. We decreased the V_T_ from 10 ml/kg to 6 ml/kg in this population, and the increase of PaCO_2_ led to a decrease in VTI_PA_/VTI_DA_, Fajardo et al. also came to the same conclusion [7]. Despite the ventilation strategies of increased PVR differed in some way, but the ultimately outcomes of lower L-R shunt was coincident. The decrease of VTI_PA_/VTI_DA_ in our results mainly attributed to the decreased L-R shunt and PA blood flow, which was also testified in another study, in which an increase in PaCO_2_ from 55 mmHg even 90 mmHg incurred statistically significant reduction in Qp/Qs [23]. Nevertheless, our modest but insignificant decrease of VTI_PA_/VTI_DA_ was the consequent of confined fluctuate range of PaCO_2_ in our study: merely 30 mmHg to 50 mmHg considering the clinical safety.

Bradely et al.demonstrated that hypoventilation improves cerebral blood flow velocity in infants with bidirectional superior cavopulmonary connection [24], and rSCO_2_ was associated with the change of cerebral blood perfusion [25]. In this study, we found increase of rSCO_2_ in accordance of increase of PaCO_2_. One reason might be that dilated cerebrovascular induced by increased PaCO_2_ [26, 27]. Another possible reason was increased CO for decrease of L-R shunt and PA flow while DA remain unchanged. It’s seems that the cerebrovascular dilation component was predominated contributor to the increased rSCO_2_.

Previous studies have suggested that hypocarbia alkalosis should be vigilant in children with elevated pulmonary artery tension due to pulmonary vasodilation [28]. But it need be cautious that although hypercarbia could induce a series of advantages, including mitigated the pulmonary over-circulation and dilated cerebral vascular bed, as indicated by decreased L-R shunt, PA blood flows and VTI_PA_/VTI_DA_ and increased rSCO_2_ in our study. Nonetheless, hypercarbia would also cause disadvantage of decreased PaO_2_ by alveolar hypoventilation. Pervious study founded systemic oxygen saturation initially improved as the Qp/Qs declined in animals modes, whereas decreased reversely after the Qp/Qs below than 0.7 [29]. Moreover, the hypercarbic probably induced an increase in heart rate, even arrhythmia and acute right heart failure which might be undesirable for the adequate oxygen supply during perioperative. As our results manifested, PaO_2_ decreased from 216.89 to 203.74 mmHg as PaCO_2_ increased, accompanied by a small rise in HR. Therefore maintaining the ratio of Qp/Qs near 1 both balance systemic blood flow and pulmonary venous oxygen content is a focus for anesthesiologists during surgery of children with L-R shunt. As seen in our results, rising PaCO_2_ from 31.51 to 44.24 mmHg decreased the VTI_PA_/VTI_DA_ absolute 0.12 and increased rSCO_2_ almost 2%, meanwhile, PaCO_2_ in 22 children were between 40 and 50 mmHg at V_T_ 6 ml/kg. Hence, maintaining PaCO_2_ between 40 and 50 mmHg by V_T_ 6 ml/kg is favorable for children with L-R shunt, in part because of reductions in L-R shunt and pulmonary blood perfusion and in part because of an increase in rSCO_2_. And it may be unwise to concentrate on minimizing L-R shunt, PA and VTI_PA_/VTI_DA_ through limitless increasing PaCO_2_, ignoring risks of pulmonary hypoperfusion [30]. Besides, consecutively monitoring EtCO_2_ could be used as a convenient method to prevent excessively high PaCO_2_ [31].

For children with defect> 10 mm, their higher VTI of PA and lower intraoperative rSCO_2_ may result from the excessive pulmonary hyperperfusion and relatively insufficient of cerebral blood flow [32]. The prolonged length of stays in defect> 10 mm was consistent with previous postoperative follow-up study, in which an increased risk of postoperative cognitive impairment and a significantly prolonged LOS in patients with low intraoperative rSCO_2_ were revealed [33]. The possible reason of the weaker vascular reactivity to higher PaCO_2_ in children with defect> 10 mm may accounted for their excessive pulmonary blood flow [34], which has been suggested produce alterations in the pulmonary vasculature, including vasoocclusive intimal thicking and medical hypertrophy [35]. Extremely patients finally evolved with obliterative pulmonary vascular disease. In previous case, children who may already been developed in pulmonary vascular obstructive disease only improved lower CO via banding the pulmonary artery, but without reactivity at a high level of PaCO_2_ [36]. Thus, we need realized that adjusting the ratio of Qp/Qs by increasing PaCO_2_ may not always be applicable to all patients, especially for children older than 18 month with defect> 10 mm. Other measures of changing PVR or SVR should take into consideration individually. Furthermore, the various pulmonary vascular response to PaCO_2_ may be used to estimate the magnitude of pulmonary hypertension and severity of disease.

One of the limitations of this explorative study is the limited range of PaCO_2_ concerning patients’ safety, which contributed to the weeny decrease in VTI_PA_/VTI_DA_ in our results didn’t achieved statistical significance. Moreover, control the enrollment age within 18 month may prevent pulmonary arteriosclerosis from affecting outcomes. Therefore, further studies are still need to evaluate the effects of hypoventilation on children of different ages and defect types with L-R shunt, and develop individualized ventilation settings to optimize the Qp/Qs under various pulmonary vascular reactivity.

## Conclusions

In conclusion, our findings recommended a feasible perioperative ventilation strategy for children with L-R shunt. That is maintaining PaCO_2_ at 40-50 mmHg by V_T_ 6 ml/kg would be helpful to mitigate the excessive L-R shunt after anesthesia, and achieve a favorable VTI_PA_/VTI_DA_ accompanied by an improvement in cerebral blood perfusion.

## Data Availability

All data generated or analyzed during this study are available from the corresponding author on reasonable request.

## Abbreviations

CHD: Congenital heart disease
DA: Descending aorta
EtCO_2_: End-tidal carbon dioxide partial pressure
IAP: Invasive arterial pressure
L-R: Left-to-Right
NIRS: Near-infrared spectroscopy
PA: Pulmonary artery
PaCO_2_: Arterial partial pressure of carbon dioxide tension
PVR: Pulmonary vascular resistance
rScO_2_: Regional cerebral oxygen saturation
TEE: Transesophageal echocardiography
V_T_: Tidal volume
VTI: Velocity time integral
